# Speciation of Tellurium During Volatilization from Lead–Bismuth Eutectic and Its Chemical Transport Properties

**DOI:** 10.1021/acs.jpcc.6c04168

**Published:** 2026-08-27

**Authors:** Vladislav Zobnin, Ana Ivan, Robert Eichler, Alexander Vögele, Dominik Herrmann, Zeynep Talip, Jörg Neuhausen

**Affiliations:** † PSI Center for Nuclear Engineering and Sciences, Paul Scherrer Institute, Forschungsstrasse 111, 5232 Villigen, Switzerland; ‡ ALFRED Project Implementation, Institute for Nuclear Research (RATEN-ICN), Câmpului Street 1, 115400 Mioveni, Romania; § Department of Chemistry, Biochemistry and Pharmaceutical Sciences, University of Bern, Freiestrasse 3, 3012 Bern, Switzerland; ∥ Department of Mechanical and Process Engineering ETH Zurich, Leonhardstrasse 21, 8092 Zurich, Switzerland

## Abstract

Tellurium (Te) volatilization from lead–bismuth eutectic (LBE) was investigated by thermosublimatography to resolve the chemical form of Te transported from a Pb-rich liquid metal and its subsequent surface-mediated transformations. For dilute Te in LBE (*x*_Te_ ≈ 10^–3^), the dominant transported species was assigned to PbTe­(g), which deposited at high temperature in both fused-silica and 316L stainless-steel columns. This indicates that lead-based coolants intrinsically suppress Te release, as Te bound in PbTe is less volatile than elemental Te. In fused-silica columns, deposited PbTe partially decomposed under more oxidizing carrier-gas conditions, causing secondary release and low-temperature deposition of elemental Te, most consistently explained by Te_2_(*g*) formation. In contrast, PbTe remained stable on 316L stainless-steel surfaces even under water-saturated conditions, consistent with local redox buffering by the steel surface. These results demonstrate that Te transport from Pb-rich liquid metal is governed not only by its chemical form, but also by gas-phase conditions and surface-mediated processes. The findings provide insight into chalcogen transport at liquid metal/material interfaces and demonstrate how surface selection and carrier-gas redox conditions can influence Te mobility.

## Graphical abstract

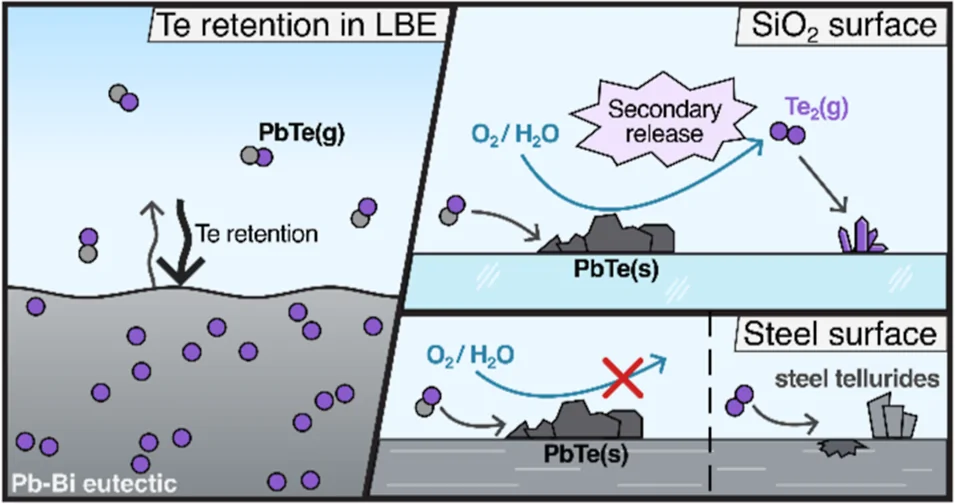

## Introduction

The development of advanced nuclear reactors cooled by heavy liquid metals (HLM), particularly lead–bismuth eutectic (LBE), has gained significant attention due to their potential for enhanced safety and sustainability. LBE combines favorable thermophysical properties with good chemical stability and is well suited to fast-reactor neutronics.^1^ In accelerator driven systems (ADS), which enable more efficient transmutation of minor actinides, it simultaneously serves also as a spallation target.

However, interactions of LBE with fission, spallation and activation products generated during operation present complex challenges for safe and reliable operation of these systems.^2–4^ Radionuclides of volatile elements are of particular concern, as they may evaporate from the coolant and accumulate in the cover gas during operation and get volatilized in beyond design accident scenarios.

Among the major fission products, tellurium (Te) is noteworthy for its volatility, chemical reactivity, and resulting implications for structural material corrosion and radiation safety. In HLM systems, Te can originate from activation of trace impurities, which, even at parts-per-million levels in large coolant inventories, can yield kilogram-scale integral quantities. In ADS, Te isotopes are also produced by high-energy proton and neutron induced fission of lead and bismuth^5,6^ and spallation. In the event of fuel cladding failure, fission-produced Te may also be released in macroscopic amounts from the fuel directly into the coolant. The fission yields of Te isotopes are significant and some of its isotopes, like ^131^Te (*T*_1/2_ = 25 m), can act as delayed sources of radioactive iodine, ^131^I (*T*_1/2_ = 8 d). Tellurium is also a chemical homologue of polonium (Po), another chalcogen and the main radiotoxic impurity formed in LBE coolants,^2,3^ making Te a useful surrogate for understanding macroscopic Po chemistry in HLM systems. Ultimately, understanding Te evaporation behavior and gas-phase speciation is essential for predicting its transport and for developing effective trapping and retention strategies.

Insights into Te transport can be drawn from studies in water-cooled reactor systems, where its behavior has been shown to be highly sensitive to environmental conditions. Analysis of Te release following the Fukushima accident concluded that its transport outside the containment was primarily associated with aerosols.^7^ Laboratory experiments likewise showed aerosol-dominated transport, strongly enhanced in oxygen- or steam-rich atmospheres.^8^ The increased volatility of TeO_2_ in steam has been attributed to the potential formation of the gaseous oxyhydroxide species TeO­(OH)_2_,^9,10^ although their existence was not unambiguously verified experimentally.

In contrast, studies of Te behavior in LBE remain comparatively limited. Experiments on the release of no-carrier-added (n.c.a.) chalcogen admixtures from LBE showed increasing volatility from selenium to Po.^11^ Early transpiration studies by Ohno, Kurata and co-workers, with Te-doped LBE demonstrated a lower Te vapor pressure compared to ideal solution behavior, which was attributed to PbTe formation; this assignment was supported by enhanced Pb evaporation in Te-containing systems but remained indirect rather than a direct identification of the gas-phase species.^12^ Reduced Te release from LBE was further confirmed by later vapor-pressure studies,^13^ whereas deposition studies using SiO_2_ filters identified PbTe and metallic Bi among the condensed phases.^14^ In addition, aerosol formation has been suggested to influence Te transport in liquid metal systems, with chemical analyses indicating oxidation of Te to the Te­(IV) state in such species.^15^ However, the available data do not provide a direct identification of the transported gas-phase species and therefore do not unambiguously distinguish transport as PbTe­(g) from possible Bi–Te species or from separate Pb- and Te-containing vapors that react only upon condensation. In addition, unlike the extensively studied interactions between LBE and structural steels, the subsequent fate of Te-containing species and deposits on structural materials has received little attention, although their stability or secondary transformation on surfaces is important for materials compatibility and for assessing capture and retention systems.

Accordingly, two important questions remain unresolved: the chemical nature of the primary Te-bearing vapor released from LBE and the stability or secondary transformation of the resulting deposits under different gas-phase and surface conditions. In this work, we apply thermosublimatography to investigate the transport and deposition of Te released from dilute solution in LBE under controlled dry and humidified He, and reducing He/H_2_ atmospheres. Temperature-resolved deposition profiles, combined with postexperimental characterization of the collected deposits, are used to probe the chemical forms present after transport and to evaluate whether transformations occur following the surface deposition. Experiments performed in fused-silica and stainless-steel columns further allow the assessment of influences of the surface material on Te behavior throughout the transport–deposition process.

## Materials and Methods

### Thermosublimatography Technique

The thermosublimatography technique is used to determine vapor composition over solid alloys and melts through evaporation/condensation equilibrium. Vapors emanating from the sample at elevated temperatures are transported along a tubular column downward an applied temperature gradient until they reach a condensation temperature where they deposit. Deposition temperatures (*T*_dep_) can be estimated from standard thermodynamic properties of the involved substances and experimental parameters using eq 1,^16^ assuming ideal gas behavior. The chemical compound deposited in the experiment can then be identified/confirmed by matching the observed *T*_dep_ with the calculated *T*_dep_ of plausible candidate substances as well as by chemical analysis. The thermochemical data used for the *T*_dep_ calculations are summarized in Table 1.

1 $$T_{dep}=\frac{\Delta_{r}H_{298}^{{}^{\circ}}}{\Delta_{r}S_{298}^{{}^{\circ}}-R\ln\left(\frac{mRT_{0}}{M\dot{V_{0}}tp^{{}^{\circ}}}\right)}$$

where *m* is the transported mass of the assumed Te species, *M* is its molar mass, $\dot{V_{0}}$ is the carrier-gas flow rate referenced to *T*_0_ and *p*°, *t* is the experiment duration, *R* is the gas constant, and Δ_r_ *H*_298_° and Δ_r_ *S*_298_° are the standard reaction enthalpy and entropy for the process written in Table 1.

**Table 1:** Thermodynamic Data Used for Calculating Deposition Temperatures^a^

process/reaction	Δ_r_ *H*_298_° (kJ mol^–1^)	Δ_r_ *S*_298_° (J mol^–1^ K^–1^)	references
Te ⇌ Te(g)	211.7	133.2	^17^
2Te ⇌ Te_2_(g)	160.4	163.2	^18^
PbTe ⇌ PbTe(g)	223.8	161.6	^17^
BiTe ⇌ BiTe(g)	217.6	175.1	^18^
TeO_2_ ⇌ TeO_2_(g)	264.0	200.9	^17^
Te + 1/2O_2_(g) ⇌ TeO(g)	74.5	88.6	^17^
Te + H_2_O(g) ⇌ TeO(g) + H_2_(g)	316.3	132.9	^17^
Te + H_2_(g) ⇌ TeH_2_(g)	99.6	48.8	^17^

a The listed values correspond to standard reaction enthalpies and entropies for the reactions as written.

Multiple depositions may indicate several species forming and simultaneously evaporating from the sample. In such cases, the masses were estimated from the total evaporated mass and the relative area of each individual deposition along the column. However, depositions at lower temperatures can also arise from depositions at higher temperature decomposing or reacting with carrier-gas components. Such effects can be assessed comparing distributions along columns after experiments of different duration or after reheating columns without starting sample.

In thermosublimatography, transport of macroscopic amounts results in deposition temperatures governed predominantly by evaporation–condensation equilibrium, so that the influence of the substrate is generally small compared with trace thermochromatography, unless chemical reactions with the surface intervene. Finite gas–surface mass transfer and heterogeneous nucleation may nevertheless cause deviations from the equilibrium deposition temperature. In this work, columns of fused silica and 316L stainless steel were used. Fused silica, widely applied in gas chemistry experiments, provides high thermal stability and chemical inertness. This material is relevant to silica-based fibers proposed for cover gas conditioning systems and to silicates which enables extrapolation to concrete used in surrounding reactor construction materials. Complementary experiments were conducted with 316L stainless steel, a standard material in nuclear technology and a candidate structural material for MYRRHA reactor components. To further examine the role of the chemical nature of the surface in Te transport, additional experiments were performed with fused-silica wool placed inside stainless-steel columns (see Section S3 of the Supporting Information). These experiments were designed to assess whether the presence of silica surfaces within a metallic environment influences the observed material-dependent behavior.

### Materials and Sample Preparation

LBE material with a nominal purity of 99.999% was supplied by SCK·CEN. According to supplier-provided neutron activation analysis data, the main impurities were Ag, Cu, and Hg, with concentrations of 18.8(9), 7.1(3), and 2.42(12) ppm_w_, respectively. All other analyzed elements were either below 1 ppm_w_ or below the detection limit of the method.

For sample preparation, LBE was reduced in a fused-silica boat within a tubular furnace using a flow of helium (He) with a 10%-vol. hydrogen (H_2_) admixture in a stepwise procedure. The process started at 600 °C and continued until a clean metal surface was achieved. The reduction was then repeated stepwise with 20–50 °C temperature decrements until reaching 430 °C.

Although Te is not expected to oxidize (PbO formation is thermodynamically preferred), LBE reduction was carried out to avoid inhomogeneities associated with residual surface oxides. Segregated PbO phases may provide interfaces for Te accumulation, affecting its distribution in the melt, as suggested also for Po.^19^

For mixing with LBE, elemental Te with natural isotopic composition (Te granules, 99.999% purity, Fluka) was activated at the PNA station of the SINQ Neutron Irradiation Facility at PSI^20^ to produce the γ-emitting isotope ^123m^Te via ^122^Te­(n, γ)^123m^Te (natural abundance of ^122^Te is 2.55%). The integral thermal neutron fluence was approximately 8×10^19^ n cm^–2^. The activated Te was stored for approximately 3 months to allow for decay of ^131^I, originating from coproduced ^131^Te. The high neutron fluence was required to achieve sufficient activity for detecting Te at its dilute concentration in LBE. For experiments with elemental Te, activation was performed at the NAA station of the same facility using a thermal neutron fluence lower by approximately 4 orders of magnitude, sufficient to obtain detectable ^123m^Te activity.

Weighed amounts of LBE and activated Te (target molar fraction of Te was 0.001, total mass ∼ 11 g) were sealed in a fused-silica ampule evacuated with a turbomolecular pump and held at 1000 °C for 24 h, with periodic shaking to ensure homogenization of the melt. The ampule was then rapidly quenched in a water bath to prevent the segregation of Te species in the LBE. This material is in the following denoted as LBE–Te. Six small fragments (6.3–56.8 mg) from both the bulk and surface of LBE–Te were dissolved in 7 M HNO_3_ and analyzed using γ-ray spectrometry to verify Te content and its distribution. All handling and storage of LBE–Te were carried out under inert atmosphere in a glovebox (H_2_O­(g) and O_2_(g) contents were below 1 ppm, MBraun). The samples were only briefly exposed to air during transfer to the experimental setup immediately before each run.

Two types of columns were used. Fused-silica columns were supplied by Schmizo AG. Stainless-steel columns (AISI 316L) were provided by Sandvik Materials Technology. According to the manufacturer, the nominal chemical composition of the steel (in wt %) is Fe (balance), Cr 16.0–18.0, Ni 10.0–14.0, Mo 2.0–3.0, with minor constituents: C, Mn, Si, P, and S.

### Experimental Setup

The thermosublimatography apparatus consisted of a horizontal tube furnace providing a controlled temperature gradient and a gas supply system enabling defined carrier-gas composition. The furnace comprised a starting zone (SF), where the sample was heated to the evaporation temperature, and a deposition zone with an approximately linear negative temperature gradient (∼13 K cm^–1^) over a length of approximately 60 cm. The temperature profile was measured under operating conditions using a thermocouple with 1 cm spatial resolution. Thermal equilibration required 30–60 min and was performed prior to sample insertion.

The sample was introduced into the hot zone using a mechanical feed-through (Supporting Information), marking the start of the experiment. Downstream of the column, a charcoal trap (CT; PFA tube filled with activated charcoal between fused-silica wool plugs) was installed to retain volatile species and aerosols.

Columns (inner diameter 5 mm) were made either of fused silica or AISI 316L stainless steel. Prior to use, they were cleaned with water, rinsed with acetone, dried in air, and conditioned under carrier-gas flow during temperature equilibration.

The carrier gas (He 5.0 or He with 10%-vol. H_2_ admixture) was supplied either directly or via a recirculating purification loop, “direct” and “loop” setups in Table 2, respectively. The loop consisted of stainless-steel tubing with Swagelok fittings and included a drying column (DC, filled with SICAPENT) and a high-temperature getter furnace (annealed Ta wire at ca. 1000 °C, ideally corresponding to an oxygen fugacity around 10^–24^ bar). The Ta getter removed O_2_ and also contributed to H_2_O removal; the operating temperature was chosen to minimize H_2_ uptake during experiments with H_2_-containing carrier gas. Gas circulation was maintained by a metal bellows pump (MBP), and flow rates were controlled by mass flow controllers (MFC; Brooks 5850S/BC) calibrated with a flow verifier (MesaLabs Defender 530+). Moisture levels were monitored using a dew point meter (DP; EasyDew, Mitchell Instruments), while gas composition, including O_2_, was monitored semiquantitatively using a mass spectrometer (MS; Cirrus2, MKS).

**Table 2:** Experimental Conditions of Performed Thermosublimatography Experiments

				preconditioning time (h)		
exp.	setup	experiment time (min)	carrier gas	SICAPENT	Ta getter	φ(H_2_O_(g)_) (ppm)
Fused-Silica Columns						
IA	loop	60	He	4.5	4.5	0.9_‑0.3_^+0.4^
IB	loop	317	He	67	2	0.03_‑0.01_^+0.02^
IIA	loop	60	He	2.75	0.2	1.6_‑0.5_^+0.7^
IIB	loop^a^	304	He	0	0	3.0_‑0.9_^+1.3^
IIIA	loop	60	He/H_2_	19.5	4	0.9_‑0.3_^+0.4^
IIIB	loop^a^	297	He/H_2_	7^b^	5	15_‑4_^+6^
IV^c^	direct	60	He	single pass	0	66_‑14_^+18^
Stainless-Steel Columns (316L)						
V	loop	110	He	4	4	0.7_‑0.2_^+0.3^
VI	direct	105	humidified He	0	0	>1 × 10^4^
VII^c^	loop	64	He	>20	4	0.03_‑0.01_^+0.02^

a PFA bypass avoiding getter and SICAPENT.

b After gettering the gas mixture, the valves were switched to a humidified PFA bypass.

c Reference experiments with elemental Te instead of LBE–Te.

### Experimental Conditions

Experimental conditions are summarized in Table 2. Experiments were conducted either under nominally inert (5.0 He) or reducing (He + 10 -%-vol H_2_) atmospheres at a constant flow rate of 50 mL·min^–1^ (273.15 K, 101.325 kPa). Two types of samples were investigated: elemental Te and LBE–Te, each studied in both fused-silica and stainless-steel columns.

The initial impurity levels in 5.0 He were approximately 3 ppm of H_2_O and 2 ppm of O_2_. In loop mode, O_2_ and H_2_O were removed by the high-temperature Ta getter, with additional H_2_O removal by the drying column. Consequently, O_2_ and H_2_O levels could not be varied independently during gas purification: increasing getter exposure simultaneously decreased the concentrations of both oxidizing impurities. The MS measurements were used to monitor relative changes in O_2_ concentration, but did not provide a quantitative O_2_ concentration during individual experiments. Intermediate humidity levels were adjusted by controlled preconditioning times, and higher moisture contents were obtained either by bypassing purification stages via PFA tube (Figure 1) or by bubbling the carrier gas through deionized and degassed water in “direct” mode. Selected experiments were also performed without getter purification. These variations in H_2_O and O_2_ contents, together with the use of reducing He/H_2_ carrier gas, were introduced to assess the sensitivity of Te transport and deposition behavior to gas-phase composition and to provide insight into the underlying chemical processes.

**Figure 1:**
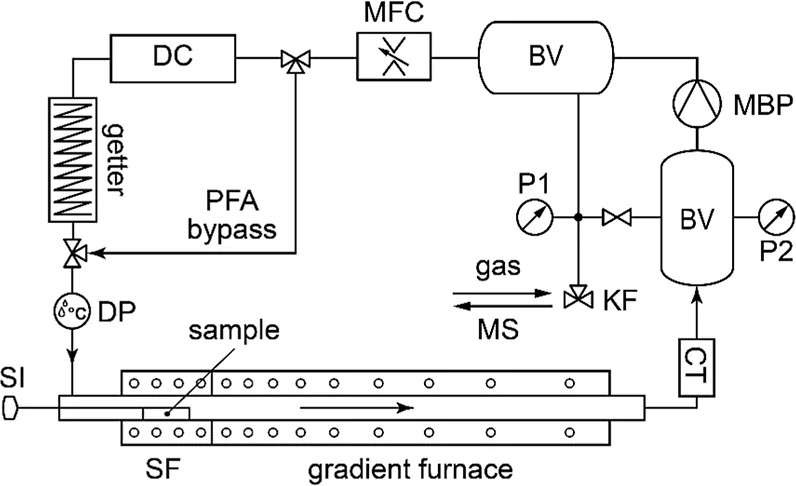
Setup scheme for thermosublimatography experiments. DC—drying column; MFC—mass flow controller; BV—buffer volume; MBP—metal bellows pump; P1, P2—pressure gauges; DP—dew point meter; MS port −6 mm tube fitting for connection to a gas line or mass spectrometer; KF–KF—flange for connection to a vacuum pump; SI—sample-insertion rod; CT—charcoal trap; SF—starting furnace.

After establishing the temperature gradient, the sample (50–200 mg) was inserted into the starting zone (ca. 820 °C). Typical experiment duration was 1 h, sufficient to establish deposition profiles under the given gas flow, evaporation temperature, and geometry conditions. In chosen experiments, longer durations were applied to study the evolution of deposition patterns. At the end of the experiment, the gas flow was stopped, the sample was remotely removed from the starting zone, and the system was cooled. The column was then extracted and analyzed for Te distribution.

To evaluate possible re-evaporation or secondary transformations, selected columns were reheated without the initial LBE–Te sample. These experiments are referred to as second-heating experiments and are paired with the corresponding first-heating experiment, i.e., the initial experiment performed with the sample present (e.g., IA/IB in Table 2).

### Characterization Techniques

Quantification of ^123m^Te was performed using a Broad Energy Germanium detector (BEGe Canberra-Mirion), using the 159 keV photopeak (*p* = 84%, *T*_1/2_ = 119.2 d). Spherical granules of activated Te (*d* ≈ 1 mm, *m* = 3.1–3.3 mg) were measured at 544 mm distance from the detector and treated as point source in activity determination. The solid residues of LBE–Te samples remaining after the experiments, as well as pieces of initial LBE–Te sample, were dissolved in 7 M HNO_3_ (4 mL) prior to the measurement to avoid self-absorption in LBE. A well-defined geometry (4 mL glass vials, inner diameter 12.8 mm, sample to detector distance 50 mm) was used for these measurements. The γ-X-ray true coincidence summing inherent to ^123m^Te measurements in this geometry was negligible (186 keV/159 keV peak areas ratio was < 0.005).

Relative distributions of Te along the columns were mapped by direct measurements of the ^123m^Te 159 keV photopeak count rate with a Pb-collimated coaxial HPGe detector in 1 cm steps. The count rate in each segment was normalized to the total ^123m^Te count rate measured along the scanned column, so that the sum over all column segments equals 100%. Thus, the yield shown in thermosublimatograms represents a relative axial distribution rather than an evaporation yield from the initial sample. Charcoal traps were measured directly on the end-cap of the detector for 7200 s for integral ^123m^Te activity.

Deposits were additionally examined by scanning electron microscopy coupled with energy-dispersive X-ray spectroscopy (SEM/EDX; Nvision 40, Zeiss) at the PSI Electron Microscopy Facility.^21^ Fused-silica fragments carrying the deposits were sputter-coated with a thin carbon layer prior to analysis, whereas stainless-steel segments were cut open to expose the inner surface and analyzed directly, without coating. Secondary electron images and point EDX spectra were acquired at accelerating voltages of 10–20 kV, selected according to the substrate and deposit morphology. EDX spectra were evaluated using Oxford INCA software and used for semiquantitative assessment of the elemental composition of selected deposits and surface regions. Because of the semiquantitative nature of EDX and possible contributions from the underlying substrate, the resulting compositions were used primarily to distinguish Pb–Te, Te-rich, Pb–Bi, and steel-reaction deposits rather than for precise phase identification.

### Data Evaluation

All reported activity uncertainties correspond to one standard deviation. Activities were obtained from peak-area analysis of HPGe γ-spectra using the Genie2000 software package (Canberra-Mirion). Activity measurements used to determine the evaporated Te mass from the sample were corrected for decay since the sample characterization date. Count rates of Te distribution along column were measured within 48 h after the experiment and therefore were not corrected for decay.

The water vapor volume fraction was calculated from the measured dew point using a saturation vapor pressure correlation for water^22^ and the total pressure used in the corresponding experiment: *p*_tot_ = 1.0 bar for the “direct” setup at atmospheric pressure and *p*_tot_ = 1.4 ± 0.1 bar for the “loop” setup experiments. Uncertainties were estimated from the dew-point accuracy of ±2 °C and the pressure-reading uncertainty by recalculating φ­(H_2_O) at the limiting values of *T*_DP_ and *p*_tot_, resulting in asymmetric errors. The uncertainty of the saturation–pressure correlation was minor in comparison.

Thermodynamic equilibrium calculations for Figure 5 were performed using the Reaction Equations module of HSC Chemistry 9 with the thermochemical database supplied with the software.^23^ Equilibrium constants were obtained for the reactions as written. Condensed phases were treated as pure phases with unit activity, and gas species were treated as ideal gases with activities expressed as *p*_*i*_/*p*°. The equilibrium expressions were rearranged to calculate the boundary *p*_H_2_O_/p_H_2__ ratios as a function of temperature. The H_2_O/H_2_ ratio represents a redox coordinate and can equivalently be expressed in terms of oxygen fugacity or oxygen chemical potential through the H_2_/H_2_O/O_2_ equilibrium. At the low total pressure employed here, gas-phase nonideality was neglected and fugacities were approximated by partial pressures. For reactions involving Te_2_(g) or TeO_2_(g), the corresponding partial pressures were fixed to literature-reported saturated vapor pressures at each temperature above condensed Te and TeO_2_.^24,25^

Temperature measurements were performed using type-K thermocouples. The deposition temperature was defined as the temperature at the position of maximum deposition, with a positional uncertainty of ±1 cm, corresponding to approximately ±15 °C. For the calculated deposition temperatures (eq 1), the dominant uncertainty contributions arise from the sublimation enthalpy and entropy, which together correspond to an estimated 1σ relative uncertainty of approximately 3.5%. Uncertainties associated with mass, flow rate, and time were not included in the error propagation, as their combined contribution is estimated to be below 1% of the contribution from the thermodynamic data and is therefore negligible (see Section S1 of the Supporting Information).

## Results

### Sample Characterization

The activity concentration of ^123m^Te in the activated Te was determined as 2.55(10)·10^8^ Bq/g at the end of bombardment. For the LBE–Te sample, the activity concentration of ^123m^Te was 8.03(6)·10^4^ Bq/g (LBE–Te) on the day of sample preparation. The Te molar fraction *x*_Te_ in the alloy was determined independently from preparation masses and γ-spectrometric activity ratios; the two approaches yielded *x*_Te_ = 9.50(3)·10^–4^, and *x*_Te_ = 9.9(4)·10^–4^, respectively. Six LBE–Te subsamples yielded activity concentrations agreeing within their individual uncertainties, with relative standard deviation among subsamples of 0.7%. Additional details on sample homogeneity are provided in Section S2 of the Supporting Information.

From the temperature dependency of Sievert’s constant for oxygen in LBE,^26^ the oxygen activity is estimated as <2.2 × 10^–4^ wt %, assuming O-saturated LBE at 430 °C. This serves as an upper limit estimate for the oxygen content of the starting material. Oxygen activity was not controlled further in the samples.

### Thermosublimatography in Fused-Silica Columns

The results of the thermosublimatography experiments in fused-silica columns are presented in Figures 2 and 3. As outlined in the experimental section, two types of experiments were conducted: a first-heating experiment with the LBE–Te sample placed at the starting position, and a second-heating experiment of the same column without starting material. In addition, carrier-gas composition was varied: nominally inert He or reducing He/H_2_ mix with different preconditioning, as summarized in Table 2.

**Figure 2:**
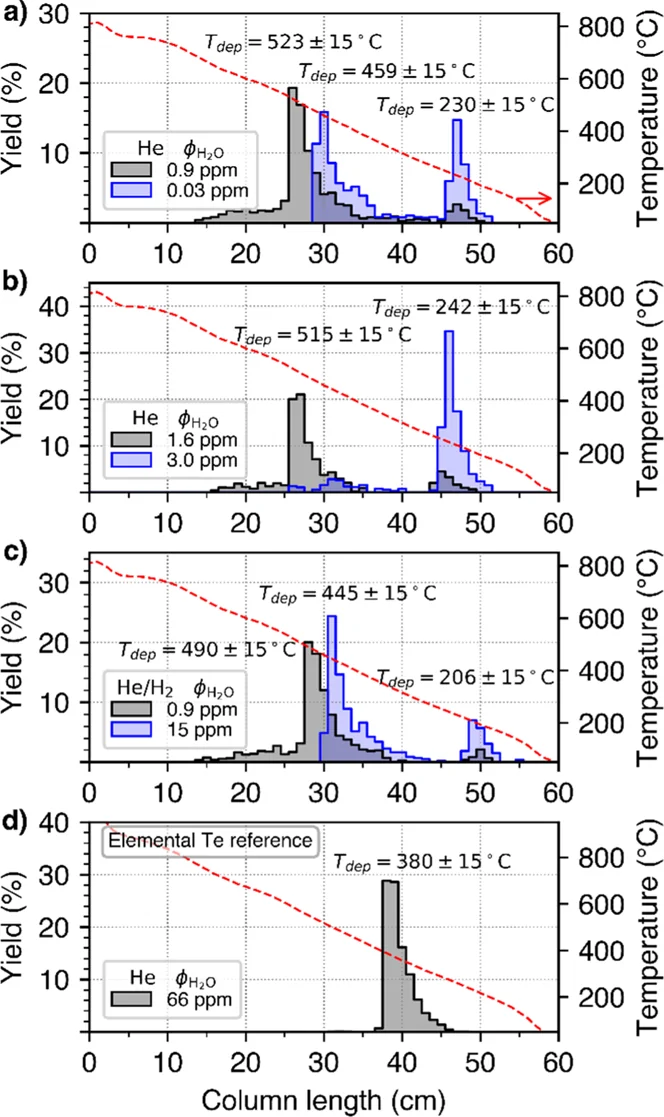
Tellurium distribution after the first-heating thermosublimatography (black histograms) and second-heating experiments (blue histograms) in fused-silica columns under varying carrier-gas conditions: experiments IA and IB (a), experiments IIA and IIB with different purification conditions during the second heating (b), experiments in reducing atmosphere IIIA and IIIB (c); and a reference experiment with elemental Te in a fused-silica column IV (d). Yield (%) denotes the relative ^123m^Te count rate in each column segment, normalized to the total count rate recovered on the column.

**Figure 3:**
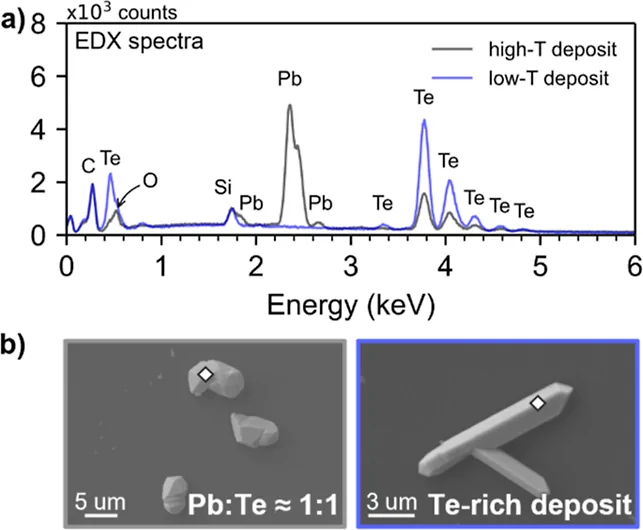
Representative SEM/EDX analysis of Te-bearing deposits formed in fused-silica columns. (a) EDX spectra acquired from the positions marked by diamonds in the SEM images. (b) SEM images of the high-temperature Pb–Te deposit and the low-temperature Te-rich deposit. The high-temperature deposit shows Pb/Te ≈ 1:1, whereas the low-temperature deposit is dominated by Te and exhibits crystalline morphology. The gray and blue frames in (b) correspond to the gray and blue spectra in (a), respectively.

Figure 2 shows the distributions of Te along the fused-silica columns after the experiments. Two deposition regions can be identified: a high-temperature region at approximately 500 °C and a low-temperature region at around 200 °C. SEM scans with EDX analysis revealed several types of deposits: Pb/Te ≈ 1:1 at the high-temperature deposition maximum, Te-rich deposits with crystalline morphology at low temperatures (Figure 3), and, in shorter runs, a broader Bi–Pb deposits containing traces of dissolved Te at higher temperatures than the Pb–Te deposition. These observations were consistent across all experiments in fused-silica columns.

The distribution of Te after the first heatings (black histograms in Figure 2) showed a similar pattern across all the experiments. After the second-heating experiments (blue histograms), the low-temperature deposition increased at the expense of the high-temperature deposition. However, despite identical experimental duration, the Te distribution after the second heating depended strongly on the carrier-gas conditioning. With higher water vapor content and no getter purification (Experiment IIB compared to IB), Te was almost quantitatively transferred to the low-temperature deposition, leaving only a small amount of PbTe (blue histogram). Figure 2c illustrates the distributions of Te in the fused-silica column after two consecutive heatings in reducing atmosphere. The results of the first heating resembled those obtained in He. During the second heating, despite water vapor levels comparable to Experiment IIB, the transfer of Te from the high-temperature to the low-temperature deposition was suppressed.

The charcoal traps located downstream of the column generally showed no or only trace amounts of ^123m^Te activity. It is not possible to determine whether transport to room temperature is associated with a specific Te chemical form or with aerosols present in the system. Slightly higher count rates in the charcoal traps were observed in experiments with H_2_-containing carrier gas.

### Thermosublimatography in Stainless-Steel Columns

Figure 4a,b shows the results of thermosublimatography experiments performed in stainless-steel columns using an LBE–Te sample in dry He and in water vapor-saturated He. In contrast to the fused-silica columns, only a high-temperature deposition region was observed. No low-temperature deposition was detected. Even under water vapor–saturated conditions, no transfer of Te to lower temperatures occurred (Figure 4b). SEM/EDX analysis revealed two types of deposits: Pb/Te ≈ 1:1 at maximum deposition and Bi–Pb deposits containing small amounts of dissolved Te, forming a smeared high-temperature shoulder of the Te distribution. No ^123m^Te activity was detected on the charcoal traps downstream of the column.

**Figure 4:**
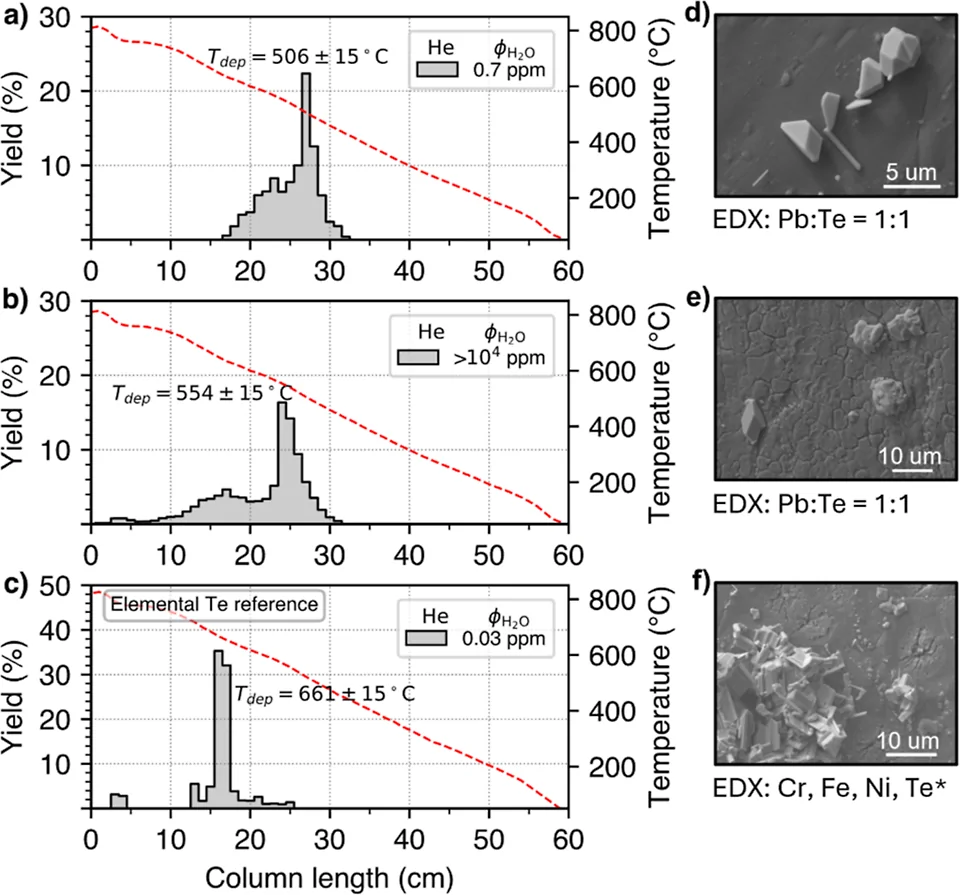
Tellurium deposition in 316L stainless-steel columns and SEM images of the deposits: thermosublimatography of LBE–Te sample in He, experiment V (a) with the corresponding SEM image of the deposit at distribution maximum (d); experiment VI with LBE–Te in humid He (b) with the corresponding SEM image (e); and thermosublimatography of elemental Te in He, experiment VII (c) with the associated SEM image (f). *Two distinct types of corrosion sites were identified, although their stoichiometry could not be reliably determined due to the steel background (see Supporting Information for details).

### Elemental Te Experiments

Experiments IV and VII with elemental Te were performed as a baseline to assist in the interpretation of the results. In both column types, a single deposition peak was observed, however, its position differed significantly between the two materials.

When elemental Te was evaporated in a stainless-steel column, deposition occurred at high temperatures (Figure 4c,f), forming tellurides of steel components. SEM/EDX analysis revealed two morphologically distinct corrosion products, namely faceted crystalline deposits and crater-like regions. Their stoichiometry could not be reliably determined due to the steel background; however, the phases were enriched in Cr, Ni, and Fe as discussed in the Section S4 of the Supporting Information. In contrast, in fused-silica columns, Te-rich deposits consistent with elemental Te were observed at lower temperatures (Figure 2d).

## Discussion

The thermosublimatography experiments revealed a clear difference in deposition behavior between fused-silica and stainless-steel columns. In fused-silica columns, two distinct deposition regions were observed, with redistribution of Te from high to low temperatures during the second heating, strongly influenced by carrier-gas conditioning. In contrast, stainless-steel columns exhibited only a single high-temperature deposition across the investigated gas conditions. Additionally, elemental Te showed markedly different behavior, reacting with steel surfaces at high temperatures while depositing at lower temperatures in fused silica. These observations indicate that both the initial chemical form of transported Te and the nature of the surface play a decisive role in its transport and deposition behavior.

### Identification of Primary Transported Species

In Table 3, the observed deposition temperatures are compared with the calculated values for the candidate species providing the closest agreement: PbTe­(g) for the high-temperature deposition and Te_2_(g) for the low-temperature deposition. Among the considered high-temperature Te-bearing species, PbTe­(g) provides the closest match to the observed deposition temperatures. The calculated *T*_dep_ values for BiTe­(g) were consistently 30–60 °C lower than observed, whereas those for TeO_2_(g) were 30–50 °C higher. Considering the combined experimental and thermochemical uncertainties, these differences do not allow BiTe­(g) or TeO_2_(g) to be excluded based on deposition temperature alone. The low-temperature deposition in fused-silica columns is most consistent with condensation controlled by the 2Te(s) ⇌ Te_2_(g) equilibrium.

**Table 3:** Observed Deposition Temperatures and Calculated Values for the Best-Matching Candidate Species in the Thermosublimatography Experiments

exp	carrier gas	*m*_evap_ Te (g)^a^	*T*_dep_ (°C) ± 15 °C	*T*_dep_ (°C) calculated
Fused-Silica Columns				
IA	He	1.314(18) × 10^–5^	523	524(28) (PbTe)
			230	247(18) (Te_2_)
IB	He	n.a	459	480(26) (PbTe)
			230	247(18) (Te_2_)
IIA	He	7.8(3) × 10^–6^	515	517(28) (PbTe)
			254	252(18) (Te_2_)
IIB	He	n.a	445	446(25) (PbTe)
			242	256(18) (Te_2_)
IIIA	He/10% H_2_	n.e	490	–
			193	
IIIB	He/10% H_2_	n.a	445	–
			206	
IV	He	3.0(3) × 10^–3^	380	393(23) (Te_2_)
Stainless-Steel Columns (316L)				
V	He	1.91(10) × 10^–5^	506	521(28) (PbTe)
VI	water-saturated He	6.48(6) × 10^–5^	554	546(29) (PbTe)
VII	He	1.56(20) × 10^–2^	661	–

a *m*_evap_ (Te) refers to evaporation from the initial LBE–Te sample. n.e. = not evaluated; n.a. = not applicable for second heating without an initial sample.

The thermosublimatographic assignment is therefore complemented by SEM/EDX analysis. The high-temperature deposition maximum showed a Pb/Te ratio close to 1:1,^27^ whereas Pb–Bi deposits containing only trace amounts of Te occurred separately at higher temperatures and the low-temperature deposits were Te-rich and exhibited crystalline morphology. Thus, the combined thermosublimatographic and SEM/EDX evidence supports PbTe as the dominant deposited phase. A minor contribution from BiTe­(g) cannot be excluded, as its lower predicted deposition temperature could result in overlap with the low-temperature tail of the dominant PbTe deposition rather than formation of a separately resolved peak.

This conclusion is also consistent with classical vapor-pressure studies of PbTe, which identify PbTe­(g) as a stable vapor species in equilibrium with condensed PbTe.^28,29^ A study on the physical vapor transport of PbTe noted that it evaporates congruently up to 850 °C, above which the vapor becomes Te-rich; however, PbTe­(g) itself is stable, with less than 1% decomposition at 870 °C.^30^ At the same time, Te_2_(g) was shown to be sensitive to the Pb/Te ratio in the condensed phase.^31^ In particular, the partial pressure of PbTe­(g) is nearly independent of this ratio within ±1 at. % from stoichiometric composition, whereas the Te_2_(g) pressure decreases strongly under Pb-saturated conditions. This is directly relevant to the present system, where Te evaporates from a Pb-rich LBE melt and the gas phase also contains evaporated Pb; both factors are expected to suppress independent Te_2_(g) formation and favor transport as chemically bound PbTe­(g). Thermodynamic comparison of gaseous PbTe­(g) and BiTe­(g) formation also favors PbTe­(g). At 1100 K, the ratio of their formation equilibrium constants is approximately 22, while the actual *p*_PbTe_/*p*_BiTe_ ratio additionally depends on the Pb/Bi activities in LBE. This further supports PbTe­(g) as the dominant species without excluding a minor BiTe­(g) contribution.

The growth of the low-temperature Te deposition at the expense of PbTe during the second-heating experiments in fused-silica columns therefore indicates secondary transformation of initially deposited PbTe rather than primary transport of elemental Te from the LBE. While the presence of additional volatile Te species cannot be completely excluded, their contribution during the initial evaporation step must be minor. The stainless-steel experiments support this interpretation, as no secondary low-temperature deposition or reaction products with steel were observed.

A further argument is provided by comparison with elemental Te transport in stainless-steel columns. If Te were transported independently from Pb, either directly from the LBE or after complete gas-phase dissociation of PbTe­(g), reaction with the steel surface would be expected, as observed for elemental Te. The absence of such reactions in LBE–Te experiments indicates that Te is transported predominantly in a chemically bound form, consistent with PbTe­(g).

### Decomposition of PbTe

In fused-silica columns, PbTe deposits are partially transformed, leading to redistribution of Te to lower temperatures during the second heating. This process was strongly enhanced under the less purified, more oxidizing carrier-gas conditions of experiment IIB compared with IB. For experiment IB, an order-of-magnitude comparison shows that the amount of Te redistributed to the low-temperature deposition, *n*(Te) = 29(3) nmol, was comparable to the cumulative amount of water vapor passed through the column, *n*(H_2_O) = 25_–9_^+14^ nmol. This shows that sufficient H_2_O was available to participate in the process, but does not establish it as the controlling oxidant. At the same time, on a cumulative gas-throughput basis an O_2_ concentration of only approximately 0.02 ppm would be stoichiometrically sufficient to account for the observed Te redistribution. Such residual O_2_ cannot be excluded because the absolute O_2_ concentration was not quantified during the experiments.

The inhibition of this redistribution in reducing atmosphere (Figure 2c) demonstrates that the process cannot be explained by hydrolysis (eq 2) but involves a redox step. If simple hydrolysis were responsible, for example by formation of Pb-hydroxide and H_2_Te as in eq 2, the equilibrium would not be influenced by the presence of H_2_. The observed behavior therefore indicates that PbTe decomposition is redox-controlled and involves an oxidative step. O_2_, H_2_O, or both may contribute oxidizing equivalents under the present conditions. The trace activities detected on charcoal traps in experiments with H_2_ nevertheless suggest that hydride formation may occur to a limited extent.

2 $$PbTe\left(s\right)+{2H}_{2}O\left(g\right)⇌Pb{\left(OH\right)}_{2}\left(s\right)+H_{2}Te\left(g\right)$$

The observed suppression of redistribution in reducing atmosphere argues against H_2_Te­(g) as the dominant transported species. Likewise, higher oxidation states, such as Te­(IV), are unlikely to dominate, as they would be expected to yield stable condensed oxide phases rather than elemental Te. The transported Te is therefore most likely present in oxidation state 0 or a low-oxidation-state Te species, such as TeO­(g),^32^ which may subsequently decompose or disproportionate to form elemental Te at the deposition site. Although hydroxide species could, in principle, contribute under humid and oxidizing conditions, their thermodynamic properties remain poorly understood and they are therefore considered only as speculative alternatives rather than definitive transport species.

Among the remaining possibilities, Te_2_(*g*) provides the most consistent explanation of the observations in this work (Table 3). For experiment IIB, the calculated deposition temperature for the 2Te(s) ⇌ Te_2_(g) equilibrium is 254 °C, in good agreement with the experimental value of 242 ± 15 °C. In contrast, the calculated deposition temperatures for Te­(g) and TeO­(g), using the equilibria listed in Table 1, differ from the experimental value by approximately 180 °C or more. The same trend was obtained for the other experiments showing low-temperature deposition.

Additionally, transport via TeO­(g), or other Te–O–H species reported only for the gas phase, would be expected to depend on the oxygen or water-vapor potential in the carrier gas.

3 $$Te\left(s\right)+\frac{1}{2}O_{2}\left(g\right)⇌TeO\left(g\right)$$

This is not observed: experiments IA and IIB show similar deposition temperatures, although IA was performed with getter-purified carrier gas whereas IIB was conducted without getter purification, so a substantially lower O_2_ level is expected in IA, although its absolute concentration was not quantified. Similarly, the comparison of experiments IB and IIB shows no clear response of the elemental-Te deposition temperature to water content, arguing against a dominant Te­(II) hydroxide transport mechanism. Together with the thermodynamically predicted deposition behavior of elemental Te in fused-silica columns, this supports Te_2_(g) as the most plausible species controlling the secondary low-temperature transport and deposition, although it was not directly identified in the gas phase. Agreement with the calculated Te_2_ deposition temperature refers to the condensation of Te_2_(g) once formed, it does not imply that its formation from PbTe proceeds at thermodynamic equilibrium. A possible net transformation consistent with these observations can be written as

$$\begin{array}{l} PbTe\left(s\right)+\frac{1}{2}O_{2}\left(g\right)\to PbO\left(s\right)+\frac{1}{2}Te_{2}\left(g\right),,\text{or}\\ PbTe\left(s\right)+H_{2}O\left(g\right)\to PbO\left(s\right)+H_{2}\left(g\right)+\frac{1}{2}Te_{2}\left(g\right) \end{array}$$ 4

The two expressions represent limiting descriptions of the oxidative transformation; the present experiments do not identify the oxygen-bearing reactant responsible for the elementary surface process.

Figure 5 evaluates the thermodynamic feasibility of selected oxidation reactions under the relevant redox conditions. At equilibrium, oxidation of Te to Te­(IV) is predicted to be more favorable than formation of elemental Te. The observed release of elemental Te therefore likely reflects a kinetically controlled, incomplete oxidation pathway, in which Te(0)-containing intermediates are formed and volatilized before further oxidation to Te­(IV) can occur. Consistently, XPS studies of oxygen-exposed PbTe surfaces at 25 °C report PbO and TeO_2_ formation,^33^ although under much more oxidizing conditions and at lower temperature than in the present experiments. At the same time, PbTe oxidation does not necessarily proceed directly to Te­(IV). Yashina et al. showed that oxygen adsorption on PbTe(001) initially produces Te(0) states associated with Te–O–Pb bonding, whereas further oxidation to Te­(IV), and ultimately PbTeO_3_, requires higher oxygen exposure.^34^ The observation of elemental Te in the present experiments may therefore reflect an incomplete, surface-mediated PbTe oxidation/decomposition step, in which Te(0) species are formed but removed by volatilization before full oxidation to Te­(IV) can occur. It is not clear whether such decomposition also occurs during PbTe transport at elevated temperatures. However, under these conditions, the PbTe transport time is rather short. Thus, complete decomposition of PbTe is likely kinetically controlled and requires longer surface retention, while continuous removal of the more volatile Te species from the PbTe deposition shifts the redox process (eq 4) toward completion.

**Figure 5:**
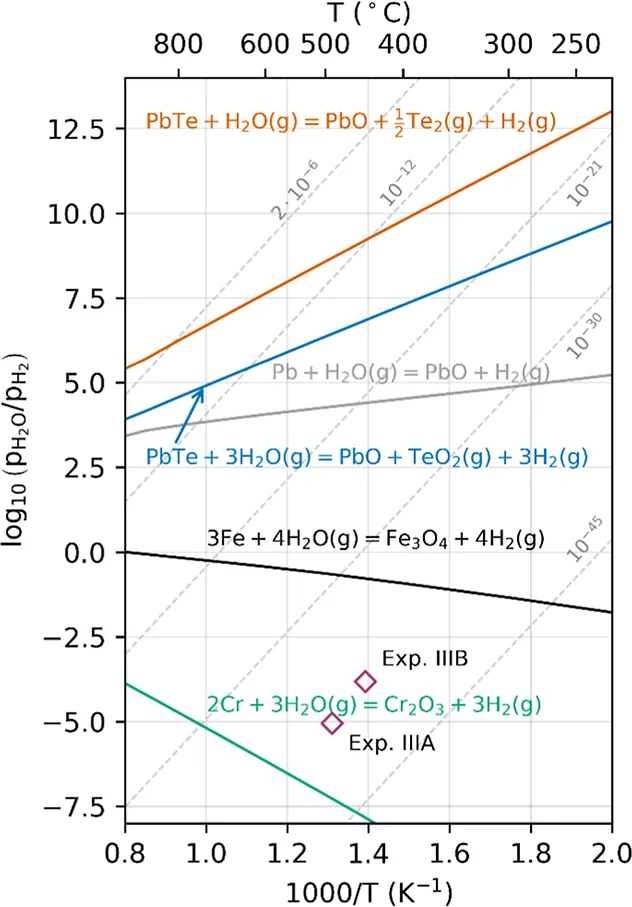
Equilibrium *p*_H_2_O_/*p*_H_2__ ratios for selected oxidation reactions as a function of temperature. The ratios were calculated from equilibrium constants obtained using the Reactions module of HSC Chemistry 9. Thermodynamic activities of condensed phases were set to 1 and for gaseous Te species they were set to the literature-reported saturated vapor pressures. Gas compositions above the respective curves correspond to conditions under which the reaction is thermodynamically favored in the direction of oxidation. Dashed reference lines indicate equivalent oxygen fugacities, *f*_O_2__, calculated from the H_2_/H_2_O/O_2_ equilibrium. The line at *f*_O_2__ = 2 × 10^–6^ bar corresponds to the nominal 2 ppm of O_2_ impurity in the supplied, nonpurified He. Conditions for the H_2_-containing experiments IIIA and IIIB are shown at their respective PbTe deposition temperatures using the measured H_2_O/H_2_ ratios.

In this context, the present results may also be viewed as a case of PbTe surface degradation under mildly reactive conditions. Most surface studies of PbTe oxidation focus on direct exposure to O_2_ and the formation of Pb/Te oxides, including Te­(IV)-containing products. In contrast, our observations show that, under mildly oxidizing carrier-gas conditions and on SiO_2_ surfaces, PbTe can undergo decomposition with formation and removal of Te species through volatilization. This pathway provides a complementary picture of PbTe instability, where degradation is governed not only by oxide formation but also by selective Te release.

### Effect of Te Dilution on Gas-Phase Speciation and Inferred Secondary Te_2_ Formation

In a real heavy-metal cooled reactor system, the Te concentration in LBE is expected to be much lower (*x*_Te_ < 10^–10^) than in the present model experiments with *x*_Te_ = 10^–3^.^35^ This lower Te activity would significantly reduce the absolute partial pressure of Te vapor species. However, it does not invalidate the proposed PbTe­(g) transport pathway, since each PbTe molecule contains only one Te atom. Thus, single-Te-containing molecular species remain relevant also under n.c.a. conditions. The present experiments, however, cannot establish whether the relative contributions of the individual Te-bearing gas species remain unchanged at such low Te activity.

Under such highly diluted cover-gas conditions, the immediate formation of Te_2_ in the gas phase is expected to be limited. Even at saturated PbTe vapor pressures in the reactor relevant temperature range of 200–400 °C, the corresponding number density (10^9^ to 10^16^ m^–3^) yields an average intermolecular spacing *d* ∼ *n* ^–1/3^ in the micrometer to millimeter range, several orders of magnitude larger than the nm-scale spacing in the cover gas at 1 bar. This makes bimolecular association of Te-containing species statistically unlikely, though, accumulations of Te upon release and redeposition from the LBE matrix at preferred deposition sites may occur. Hence, only a small amount of deposited PbTe is required to achieve significant surface coverage: based on the cubic lattice parameter of PbTe (*a* ≈ 6.5 Å), a compact monolayer corresponds to ∼10^14^ to 10^15^ units cm^–2^, i.e., on the order of 0.1 μg cm^–2^. Thus, even subμg per cm^2^ accumulation of PbTe at cold deposition sites or on filter surfaces may provide sufficient local surface coverage for neighboring Te atoms or Te-containing units to interact. Under such conditions, secondary transformation and revolatilization as polytellurium species, like Te_2_, may become possible.

The extrapolation of the present deposition behavior to reactor-relevant Te concentrations is qualitative. At very low Te fluxes, a macroscopic condensed phase may not form, and adsorption/desorption, heterogeneous nucleation, and wall retention may become more important than bulk condensation equilibrium. Consequently, the present experiments do not establish the absolute deposition temperatures, transport rates, or extent of secondary Te transformation at *x*_Te_ ∼ 10^–10^. They instead identify PbTe­(g) as the most plausible primary carrier under the investigated Pb-rich conditions and a possible surface-mediated route to secondary Te_2_ formation after local accumulation; both aspects require verification in the n.c.a. regime.

### Surface Effects on PbTe Stability

The contrasting behavior of PbTe in fused-silica and stainless-steel columns shows that its stability is controlled not only by the bulk gas composition, but also by the nature of the surface on which it is deposited. In fused-silica columns, more oxidizing carrier-gas conditions promote decomposition of PbTe and the appearance of a low-temperature Te deposition. In stainless-steel columns, by contrast, PbTe remains stable even in water-saturated carrier gas, and no corresponding low-temperature Te transport is observed.

Because PbTe decomposition on fused silica was shown to involve an oxidative step, we propose that its absence in stainless-steel columns may result from local redox buffering by the steel surface. At elevated temperature, Fe and especially Cr in stainless steel are stronger oxygen getters than Pb- or Te-containing deposits (Figure 5). Oxidizing impurities reaching the steel surface can therefore be consumed by oxidation of the alloy; in the case of H_2_O, this is accompanied by H_2_ formation, thereby lowering the local oxidation potential near the deposit toward equilibria controlled by steel oxidation. As a result, even if the bulk carrier gas contains oxidizing impurities, the immediate surface environment may remain insufficiently oxidizing for PbTe decomposition and subsequent Te release. This is plausible given the relatively large steel surface area (ca. 8 cm^2^ for a 5 cm deposition zone in a 5 mm i.d. column) relative to the submg amount of deposited PbTe.

Fused silica, in contrast, may not only fail to provide such reductive protection, but may also promote PbTe decomposition under oxidizing conditions. One possible explanation is that the fused-silica surface provides hydroxylated adsorption sites. Water adsorption, silanol formation, and rehydroxylation of siloxane bridges are well-established features of silica surface chemistry,^36^ and such sites may promote interfacial contact between PbTe-derived species and H_2_O. The observed PbTe decomposition in fused-silica columns may therefore reflect a surface-assisted process. However, because O_2_ and H_2_O were not varied independently, the present experiments do not establish a specific H_2_O-mediated role of the silica surface. Experiments with fused-silica wool inserted into steel columns, both around the sample and at the expected PbTe deposition zone, did not induce PbTe decomposition (see Section S3 of the Supporting Information). Thus, although these results do not completely rule out an active role of the silica surface, such effects appear insufficient in the presence of the surrounding steel surface. These observations are consistent with the proposed local redox-buffering mechanism, although they do not demonstrate it directly.

Similar behavior has been reported for Po in transpiration experiments, where a volatile fraction was observed in fused-silica columns but not in stainless-steel-lined columns.^37^ It was suggested that the steel surface captures the volatile Po compound. Extrapolating our observations of PbTe stability on steel surfaces to Po suggests that volatile species may not be forming at all within steel surface vicinity.

### Reactivity of Te and PbTe toward Steel

Compatibility of structural steels with LBE is strongly influenced by oxygen activity. Insufficient oxygen can promote dissolution corrosion, whereas controlled oxygen conditions favor the formation of protective oxide layers; alloy modification and protective coatings have therefore been widely investigated to improve steel compatibility with LBE.^38,39^ While these processes concern direct contact between steel and the liquid metal, the chemical form of volatile Te-bearing species represents an additional materials-compatibility factor for surfaces exposed to the gas phase.

Experiments with elemental Te in steel columns (Figure 4c,f) showed direct reaction with the steel surface at high temperatures, forming two morphologically distinct telluride phases. This behavior is consistent with thermodynamic calculations, which predict telluride formation with the main steel components, particularly Cr, Ni, and Fe, under the estimated Te partial pressure (see Table S2).

In contrast, no comparable interaction was observed in LBE–Te experiments. Instead, Te was deposited as PbTe, with its transport behavior being most consistent with PbTe­(g), without formation of tellurides with steel, indicating its significantly lower reactivity toward steel than of elemental Te. This is supported by the calculations for reactions between the proposed transported species, PbTe­(g), and steel components (see Table S4), after correction for the saturated PbTe­(g) partial pressure. Telluride formation is not expected for most considered reactions and remains only thermodynamically possible for selected Cr- and Mn-containing phases. This reduced reactivity is favorable from the perspective of materials compatibility and shows that, in addition to oxygen control at the liquid-metal/steel interface, the chemical form of Te can influence its interaction with structural surfaces after volatilization from LBE.

This conclusion should be understood within the boundary conditions of the present experiments. Direct PbTe-metal contact at elevated temperatures can lead to interfacial reactions and mutual diffusion, as reported for PbTe–Ni and PbTe–Fe systems.^40^ Thus, the absence of visible telluride formation here does not rule out PbTe–steel interaction altogether. It indicates, however, that under the low partial pressures and gas-phase transport conditions of these experiments, Te, most likely in telluride form, reacts with steel components much less readily than elemental Te.

## Conclusions

Thermosublimatography experiments strongly support the assignment of PbTe­(g) as the predominant Te-bearing species transported from LBE, with deposition occurring at high temperatures in both fused-silica and stainless-steel columns. In fused-silica systems, PbTe is partially decomposed, leading to transport of more volatile Te species and subsequent deposition at lower temperatures, likely via Te_2_(g). PbTe decomposition was favored under more oxidizing carrier-gas conditions and suppressed under reducing H_2_-containing conditions. Because O_2_ and H_2_O were not varied independently, their individual contributions cannot be resolved from the present experiments.

In contrast, PbTe remains stable in stainless-steel columns even under water-saturated conditions, and no low-temperature deposition is observed. Experiments with elemental Te showed direct reaction with steel surfaces at high temperatures, forming tellurides, whereas no such interaction was observed for LBE–Te systems. This demonstrates that PbTe is significantly less reactive toward steel than elemental Te.

The results indicate that the transport and deposition behavior of Te is governed by a combination of its chemical state, gas-phase conditions, and surface-mediated processes. The relatively low volatility of PbTe should lead to a reduced Te vapor pressure in the cover gas compared to ideal-solution assumptions for Te in LBE, suggesting that Pb-rich liquid metal inhibits Te release. In addition, PbTe exhibits lower corrosivity toward structural materials than elemental Te, which is favorable from a materials compatibility perspective. At the same time, the observed decomposition of PbTe under mildly oxidizing conditions highlights the importance of the cover gas conditioning and carefully selecting trapping materials, as surfaces such as silica may promote the formation and transport of more volatile Te species. Although gas-phase association of Te species into Te_2_ is unlikely under dilute conditions, subμg cm^–2^ surface loadings of PbTe may locally enhance the effective Te concentration and facilitate the inferred formation of Te_2_(g) on retaining surfaces. Beyond LBE-cooled nuclear systems, this finding is also relevant to the broader surface chemistry of PbTe as a thermoelectric and semiconductor material, indicating that mildly oxidizing environments may promote surface-mediated PbTe degradation and Te redistribution.

Future work should focus on quantitative Te-release data and on the mechanism of secondary Te formation. Vapor-pressure measurements of Te species above LBE are needed to determine release fractions under reactor-relevant conditions and to support source-term assessments. Extending this study toward n.c.a. Te concentrations would clarify whether secondary Te release persists in the ultradilute regime relevant to fission-product inventories. In parallel, kinetic studies of PbTe decomposition and re-evaporation, would help distinguish its transformation mechanism. Experiments with independently controlled H_2_O/H_2_/O_2_ ratios and well-defined surface materials or coatings would further allow the surface-mediated conditions governing PbTe stability to be mapped more quantitatively.

## Supplementary Material



## Data Availability

The data supporting the findings of this study are available within the article and its Supporting Information. Additional raw data are available from the corresponding author upon reasonable request.

## Notes

Supporting Information is available and includes uncertainty and sensitivity analysis for the calculated deposition temperatures, assessment of LBE–Te sample homogeneity, thermosublimatography experiments in stainless-steel columns containing fused-silica wool, and supplementary thermodynamic calculations and characterization of Te and PbTe interactions with stainless steel (PDF)
