# Supplementary material for: Speciation of Tellurium During Volatilization from Lead–Bismuth Eutectic and Its Chemical Transport Properties
Source: J Phys Chem C Nanomater Interfaces. 2026 Aug 27;130(36):12757–68. doi: 10.1021/acs.jpcc.6c04168 (PMC13573818; doi:10.1021/acs.jpcc.6c04168)
Supplement: Supplementary file 1 [file jp6c04168_si_001.pdf]

Supporting Information to “Speciation of Tellurium during Volatilization from Lead–Bismuth Eutectic and Its Chemical Transport Properties”

Vladislav Zobnin[1,3], Ana Ivan[2], Robert Eichler[1,3,4], Alexander Vögele[1], Dominik Herrmann[1], Zeynep Talip[1], Jörg Neuhausen[1*]

[1]PSI Center for Nuclear Engineering and Sciences, Forschungsstrasse 111, 5232 Villigen PSI, Switzerland

[2]ALFRED Project Implementation, Institute for Nuclear Research (RATEN-ICN), Câmpului Street 1, 115400 Mioveni, Romania

[3]Department of Chemistry, Biochemistry and Pharmaceutical Sciences, University of Bern, Freiestrasse 3, 3012 Bern, Switzerland

[4]Department of Mechanical and Process Engineering ETH Zurich, Leonhardstrasse 21, 8092 Zurich, Switzerland

*Corresponding author: joerg.neuhausen@psi.ch

Contents

S1. Uncertainty and sensitivity analysis of calculated deposition temperatures 2

S2. Sample homogeneity 2

S3. Thermosublimatography in steel columns with fused silica wool 3

S4. Te and PbTe interaction with steel 4

References 7

S1. Uncertainty and sensitivity analysis of calculated deposition temperatures

The deposition temperatures T_{dep} were calculated according to:

$$T_{dep} = \frac{\Delta_r H^\circ_{298}}{\Delta_r S^\circ_{298} - R \ln\left(\frac{m \cdot R \cdot T_0}{M \cdot \dot{V}_0 \cdot t \cdot p^\circ}\right)} \quad (1)$$

A sensitivity analysis was performed to assess the propagation of uncertainties. Relative sensitivities (S_x) were obtained from partial derivatives of T_{dep} with respect to each parameter, normalized to yield dimensionless coefficients.

$$S_x = \frac{\partial T_{dep}}{\partial x} \cdot \frac{x}{T_{dep}} \quad (2)$$

The contribution of each parameter to the relative uncertainty of T_{dep} was estimated as the product of its relative sensitivity and relative standard deviation (RSD), and the combined uncertainty was calculated assuming independent contributions.

The RSD values were assigned as follows: uncertainties for $\Delta_r H^\circ_{298}$ and $\Delta_r S^\circ_{298}$ were taken from literature data; for the flow rate $\dot{V}_0$, a conservative estimate exceeding manufacturer specifications was applied; for the time t, an experimental estimate was used; and for the transported mass m, the uncertainty was derived by propagation of γ-spectrometric measurement uncertainties.

The results are summarized in Table S1. The analysis shows that the uncertainty of T_{dep} is dominated by $\Delta_r H^\circ_{298}$ and $\Delta_r S^\circ_{298}$, while the contributions of m, $\dot{V}_0$, and t are negligible. This behavior is attributed to the logarithmic dependence of these parameters, which significantly reduces their influence.

Table S1. Sensitivity coefficients, relative uncertainties, and resulting contributions to the uncertainty of calculated deposition temperatures

Parameter	Rel. sensitivity	Max RSD	Impact (sensitivity*RSD)
$\Delta_r H^\circ_{298}$	1.00	0.0300	0.0300
$\Delta_r S^\circ_{298}$	−0.59	0.0300	−0.0176
$\dot{V}_0$	−0.03	0.0500	−0.0015
m	0.03	0.1100	0.0033
t	−0.03	0.0050	−0.0002

Including all parameters results in a relative uncertainty of T_{dep} of approximately 3.5%, while restricting the analysis to $\Delta_r H^\circ_{298}$ and $\Delta_r S^\circ_{298}$ yields an essentially identical value. Therefore, the uncertainty of T_{dep} is governed primarily by the thermodynamic data, and the contributions of experimental parameters can be neglected without loss of accuracy.

S2. Sample homogeneity

Accurate estimation of Te evaporation requires precise knowledge of the initial Te inventory in each sample. As this is derived from the bulk composition ($m(sample) \cdot x_{Te}$), a uniform distribution of Te among subsamples is essential for the validity of this approach.

The activity concentration of ^{123m}Te in LBE–Te was obtained from six individual subsamples. Each subsample was measured 1–3 times by γ-spectrometry, and the activity per unit mass (A/m) was calculated from the mean activity and the measured sample mass, with uncertainties propagated accordingly. The final value was determined as the inverse-variance weighted mean of the individual subsample results. The individual subsample results and their uncertainties are shown in Fig. S1.

The subsample results were statistically consistent within their individual uncertainties, as confirmed by a reduced chi-square of $\chi^2/\nu = 0.77$ ($\nu = 5$). This indicates that the observed scatter is fully explained

by measurement uncertainty and confirms homogeneous distribution of Te in the LBE matrix. Any residual inhomogeneities are likely associated with microcrystalline domains, and their influence is expected to decrease with increasing sample mass, approximately proportional to $\sqrt{m}$.

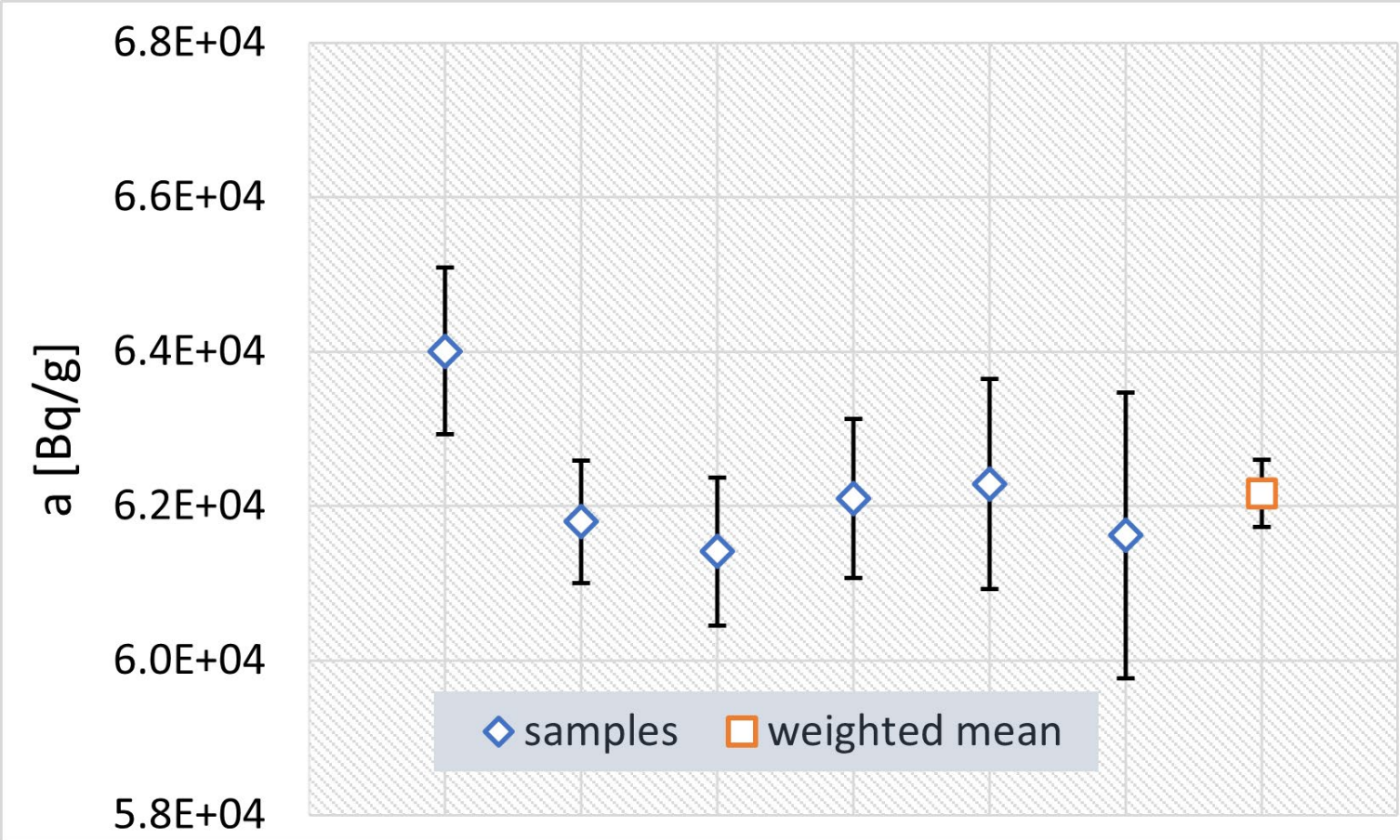

Figure S1. Te content in six sub-samples and calculated weighted mean for the LBE–Te sample

S3. Thermosublimatography in steel columns with fused silica wool

Thermosublimatography experiments with fused-silica wool inserted into 316L stainless-steel columns were performed to assess whether silica surfaces can promote PbTe decomposition under conditions where the surrounding column wall is stainless steel. This question was motivated by the different behavior observed in fused-silica and stainless-steel columns, and by the well-established interaction of silica surfaces with water. Water adsorption on silanol groups, hydroxylation/rehydroxylation of siloxane bridges, and the formation of hydroxylated surface sites are central features of silica surface chemistry [S1]. In addition, contact between water vapor and silicate surfaces can lead to formation of reactive oxygen species (ROS) [S2]. These observations motivated experiments in which fused-silica wool was placed either near the expected PbTe deposition zone or directly around the LBE–Te sample, where any surface-assisted interaction between PbTe-derived species, water, and silica would be expected to be most pronounced. Experiments were conducted under controlled carrier-gas conditions, including dry He and water-saturated atmospheres. The axial distribution of Te was determined by γ-scanning, and selected deposition regions were analyzed by SEM/EDX.

In all experiments, Pb–Te deposits were observed at temperatures close to the calculated deposition temperatures. The presence and position of fused-silica wool did not produce any systematic shift in deposition temperature or Te distribution. SEM/EDX analyses consistently identified PbTe as the dominant condensed phase, accompanied by Bi–Pb deposits containing dissolved Te. No evidence of elemental Te formation or Te–steel reaction products was detected, even under humid carrier-gas conditions.

These observations indicate that, close to the stainless-steel surface, PbTe remains chemically stable during transport and deposition. Fused-silica wool alone does not induce detectable PbTe decomposition and does not chemically retain Te under these conditions (Fig. S2a). Its role appears to be limited to physical trapping, without preventing re-evaporation. Therefore, even if hydrated silica surfaces can provide reactive adsorption sites or promote ROS-related surface chemistry, such effects are insufficient to induce detectable PbTe decomposition in the presence of the surrounding stainless-steel surface. This observation is consistent with, but does not directly demonstrate, the proposed local redox-buffering mechanism.

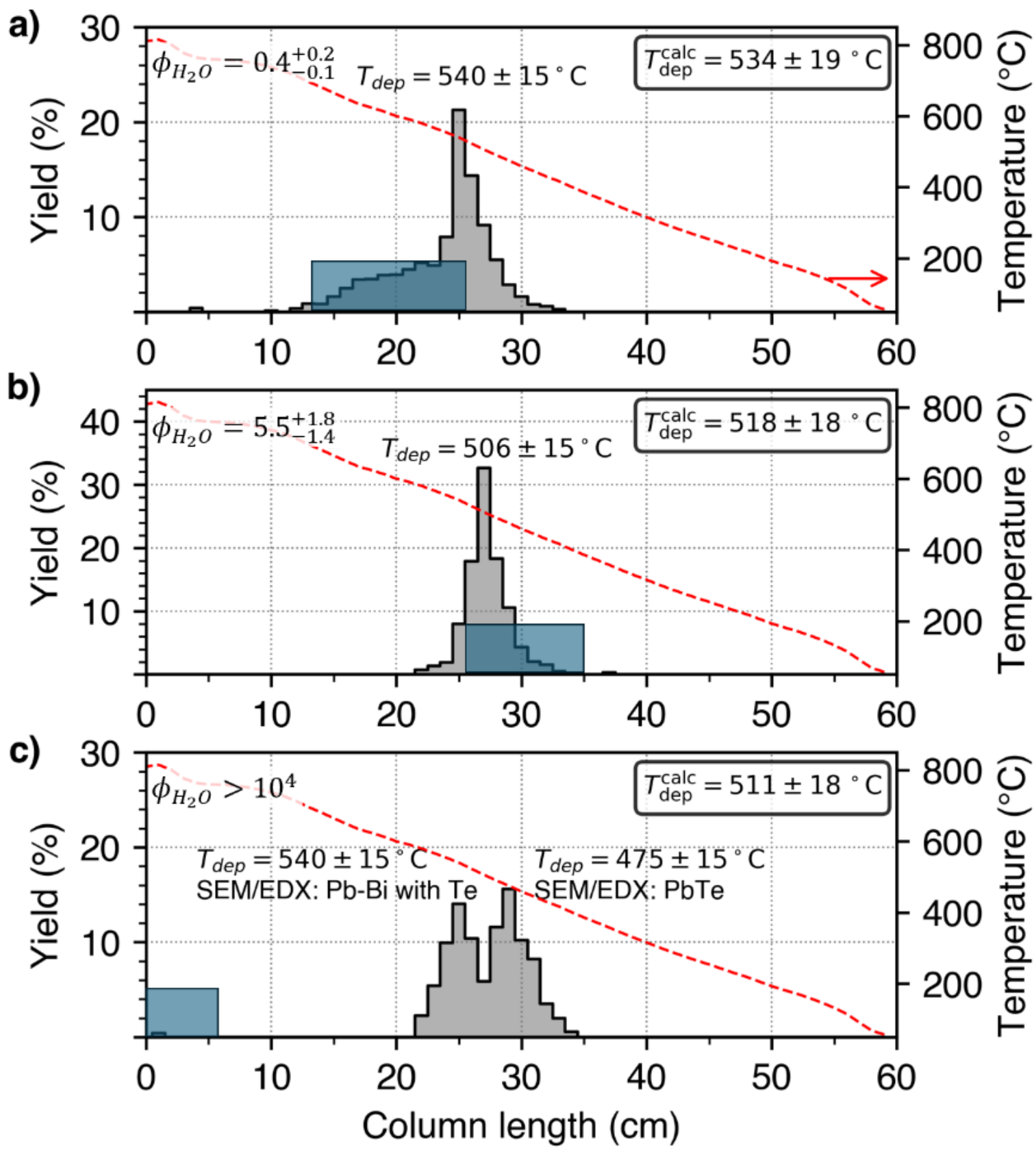

Figure S2. Thermosublimatograms of Te transported from LBE in 316L columns with fused silica wool. The axial distribution of ^{123m}Te activity is shown together with the temperature gradient along the column. The positions of the fused silica wool are indicated with blue rectangles. Deposition maxima correspond to PbTe condensation and agree with calculated deposition temperatures.

S4. Te and PbTe interaction with steel

Thermodynamic driving forces for the interaction of elemental Te with stainless-steel components were evaluated by calculating Gibbs energies of formation for metal tellurides of steel components. Standard Gibbs energies, ΔG°(T), were obtained using the Reaction Equations module of HSC Chemistry 9 [S3].

To account for experimental conditions, Gibbs energies were corrected for the estimated Te partial pressure according to:

$$\Delta G\big(T, p(Te_2)\big) = \Delta G^\circ(T) - \nu RT \ln p_{\mathrm{Te}_2}\,, \tag{3}$$

where ν is the stoichiometric coefficient of Te_2(g), and the partial pressure of Te_2(g) was estimated from tabulated vapor-pressure data for pure Te [S4]. In a further approximation, activities of metallic components in 316 stainless steel were taken into account (*–RTln a(M)*) based on literature data [S5]. All reactions were formulated per one mole of metal consumed, allowing direct comparison of the relative reactivity of individual alloy components toward Te.

The calculated Gibbs energies indicate that the formation of several telluride phases is thermodynamically favorable under the considered conditions. Among the evaluated reactions, the most negative Gibbs energies are obtained for Cr, Mn, Ni, and Mo tellurides, indicating a strong thermodynamic driving force for their formation. In particular, chromium tellurides exhibit the most negative Gibbs energies among the major alloying elements, demonstrating a pronounced tendency for preferential interaction of Te with Cr in the steel matrix. Among tellurides with near 1:1 stoichiometry, MnTe and $NiTe_{1.1}$ show the strongest thermodynamic driving force, while more Te-rich phases such as Cr_2Te_3, Ni_2Te_3, $MnTe_2$, and Mo_3Te_4 are additionally stabilized under conditions of elevated Te chemical potential.

Table S2. Calculated Gibbs energies for the formation of metal tellurides of steel components. Standard Gibbs energies ΔG°(T = 660 °C) were obtained from HSC Chemistry 9. The third column shows Gibbs energies corrected for the estimated Te_2(g) partial pressure, and the fourth column additionally accounts for the activities of alloy components.

Reaction	ΔG°(T) (kJ mol^{-1})	ΔG(T, p(Te_2)) (J mol^{-1})	ΔG(T, p(Te_2), a(M)) (J mol^{-1})
Fe + Te_2(g) = $FeTe_2$	−67.001	−3.63E+04	−3.47E+04
Fe + 0.45 Te_2(g) = $FeTe_{0.9}$	−39.825	−2.60E+04	−2.44E+04
Fe + 0.555 Te_2(g) = $FeTe_{1.11}$	−38.184	−2.11E+04	−1.96E+04
Cr + 0.75 Te_2(g) = 0.5 Cr_2Te_3	−171.348	−1.48E+05	−1.39E+05
Cr + 0.662 Te_2(g) = 2.323 $Cr_{0.43}Te_{0.57}$	−102.161	−8.18E+04	−7.28E+04
Ni + 0.5 Te_2(g) = NiTe	−53.723	−3.84E+04	−3.36E+04
Ni + 0.55 Te_2(g) = $NiTe_{1.1}$	−68.634	−5.17E+04	−4.69E+04
Ni + Te_2(g) = $NiTe_2$	−96.984	−6.63E+04	−6.15E+04
Ni + 0.333 Te_2(g) = 1.666 $Ni_{0.6}Te_{0.4}$	−50.108	−3.99E+04	−3.51E+04
Ni + 0.75 Te_2(g) = 1.5 $Ni_{0.667}Te$	−81.238	−5.82E+04	−5.34E+04
Ni + 0.55 Te_2(g) = 1.1 $Ni_{0.909}Te$	−66.943	−5.01E+04	−4.53E+04
Ni + 0.75 Te_2(g) = 0.5 Ni_2Te_3	−81.203	−5.82E+04	−5.34E+04
Mo + Te_2(g) = $MoTe_2$	−89.808	−5.91E+04	−3.81E+04
Mo + 0.663 Te_2(g) = 2.326 $Mo_{0.43}Te_{0.57}$	−83.123	−6.28E+04	−4.18E+04
Mo + 0.666 Te_2(g) = 0.333 Mo_3Te_4	−62.800	−4.23E+04	−2.14E+04
Mn + 0.5 Te_2(g) = MnTe	−134.583	−1.19E+05	−8.31E+04
Mn + Te_2(g) = $MnTe_2$	−154.588	−1.24E+05	−8.77E+04

These results indicate that Te-induced corrosion of stainless steel is governed by selective interaction with specific alloy components, with Cr being particularly susceptible, while Fe, as the major component of the steel, shows the lowest thermodynamic driving force for telluride formation among the principal alloying elements.

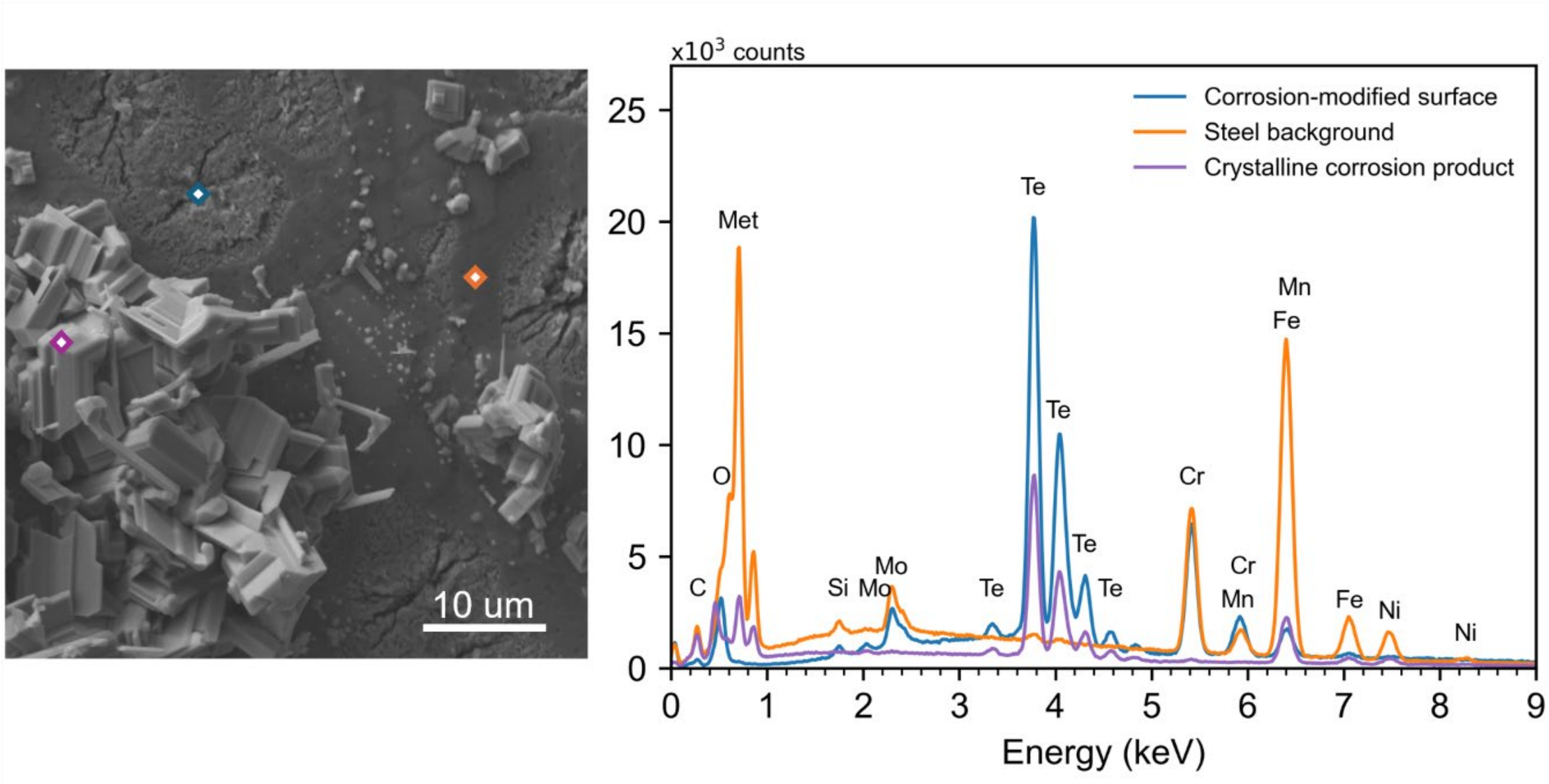

Figure S3. Corrosion of stainless steel induced by elemental Te at the deposition maximum in stainless-steel columns. Two distinct Te-induced morphological features are observed: crater-like surface regions and faceted crystalline deposits.

The corrosion of the steel surface is characterized by two distinct morphological features (Fig. S3). The crater-like regions indicate localized interaction between Te and the steel surface, associated with

material degradation and compositional redistribution. In addition, faceted crystalline deposits are observed, corresponding to the formation of discrete telluride phases.

Elemental compositions obtained by EDX analysis for representative regions are summarized in Table S3. The corrosion-modified surface exhibits a pronounced enrichment in Cr, Mn and Te, while Fe and Ni are significantly depleted compared to the bulk steel composition. This observation is consistent with the thermodynamic prediction of preferential Cr and Mn tellurides formation.

In contrast, the faceted crystalline corrosion products are dominated by Fe, Ni, and Te, with only minor Cr. Their metal-to-tellurium ratio is broadly compatible with a metal-rich telluride composition, such as an M_3Te_2-type phase with M = Fe and Ni. Similar faceted/blocky telluride morphologies have been reported for Te-vapor corrosion of Ni–Cr–Mo alloys, where Ni_3Te_2, CrTe, and $MoTe_2$ were identified by XRD [S6]. However, in the present case the phase assignment remains tentative because no crystallographic information is available and the EDX analysis is semi-quantitative.

Table S3. Elemental composition of selected regions from Fig. S3 obtained by EDX analysis (at.%).

Spectrum	O	Si	Cr	Mn	Fe	Ni	Mo	Te	C
Steel background	3.3	0.5	20.3	1.6	58.2	12.3	1.7	0.5	1.7
Corrosion-modified surface	8.0	0.7	30.9	7.2	3.0	−	2.1	44.0	4.0
Crystalline corrosion product	3.5	0.0	1.0	0.0	44.7	13.3	0.0	35.8	1.7

Overall, the SEM/EDX results show that elemental Te reacts readily with 316L stainless steel, producing localized Te-induced corrosion and chemically distinct telluride-rich products. The Cr/Mn enrichment in the corrosion-modified regions supports the thermodynamic prediction of preferential reaction with Cr- and Mn-containing steel components, whereas the faceted crystals indicate additional formation of Fe–Ni-rich telluride products. Therefore, the data demonstrate strong chemical interaction between elemental Te and steel, but do not allow unambiguous identification of individual telluride phases without complementary XRD, EBSD, or TEM analysis.

To assess whether PbTe(g) can react with stainless-steel components, analogous HSC calculations were performed for telluride formation reactions using PbTe(g) as the Te bearing reactant. The reactions were written per one mole metal consumed. Standard Gibbs energies were first calculated at $T = 660$ °C, and then corrected for saturated partial pressure of PbTe(g) (Eq. 4) [S7]. In further approximation, activities of alloy components were included.

$$\Delta G \approx \Delta G^{\circ}(T) - \nu RT ln\, p_{PbTe} - RT ln\, a(M) \quad (4)$$

Standard Gibbs energies of telluride formation from PbTe(g) are negative for all examined reactions. However, after correction for the saturated PbTe(g) partial pressure, most reactions are no longer expected to proceed from a thermodynamic perspective. At 660 °C, only formation of selected chromium- and manganese-containing tellurides remains thermodynamically possible, indicating that PbTe(g) should be substantially less reactive toward steel components than elemental tellurium under comparable conditions.

This agrees with the experimental observation that, in LBE–Te experiments, Te did not measurably react with the stainless-steel column but was transported further along the temperature gradient and deposited as PbTe according to its sublimation/condensation equilibrium. Although the formation of some Cr- and Mn-tellurides may be thermodynamically possible at the highest temperatures, the absence of such products suggests that the process is kinetically limited under the experimental conditions.

Table S4. Calculated Gibbs energies for the formation of metal tellurides of steel components in reaction with PbTe(g). Standard Gibbs energies, ΔG°(T = 660 °C), were obtained from HSC Chemistry 9. The third column shows Gibbs energies corrected for the saturated PbTe(g) partial pressure, and the fourth column additionally accounts for the activities of alloy components.

Reaction	ΔG°(T) (kJ mol^{-1})	ΔG(T, p(Te_2)) (J mol^{-1})	ΔG(T, p(Te_2), a(M)) (J mol^{-1})
Fe + 2 PbTe(g) = $FeTe_2$ + 2 Pb	−84.818	7.78E+04	7.94E+04
Fe + 0.9 PbTe(g) = $FeTe_{0.9}$ + 0.9 Pb	−47.843	2.53E+04	2.69E+04
Fe + 1.11 PbTe(g) = $FeTe_{1.11}$ + 1.11 Pb	−48.073	4.22E+04	4.38E+04
Cr + 1.5 PbTe(g) = 0.5 Cr_2Te_3 + 1.5 Pb	−184.711	−6.27E+04	−5.37E+04
Cr + 1.326 PbTe(g) = 2.326 $Cr_{0.43}Te_{0.57}$ + 1.326 Pb	−114.101	−6.27E+03	2.72E+03
Ni + PbTe(g) = NiTe + Pb	−62.632	1.87E+04	2.35E+04
Ni + 1.1 PbTe(g) = $NiTe_{1.1}$ + 1.1 Pb	−78.433	1.10E+04	1.58E+04
Ni + 2 PbTe(g) = $NiTe_2$ + 2 Pb	−114.801	4.78E+04	5.26E+04
Ni + 0.666 PbTe(g) = 1.666 $Ni_{0.6}Te_{0.4}$ + 0.666 Pb	−56.041	−1.88E+03	2.92E+03
Ni + 1.5 PbTe(g) = 1.5 $Ni_{0.667}Te$ + 1.5 Pb	−94.601	2.74E+04	3.22E+04
Ni + 1.1 PbTe(g) = 1.1 $Ni_{0.909}Te$ + 1.1 Pb	−76.742	1.27E+04	1.75E+04
Ni + 1.5 PbTe(g) = 0.5 Ni_2Te_3 + 1.5 Pb	−94.566	2.74E+04	3.22E+04
Mo + 2 PbTe(g) = $MoTe_2$ + 2Pb	−107.625	5.50E+04	7.60E+04
Mo + 1.326 PbTe(g) = 2.326 $Mo_{0.43}Te_{0.57}$ + 1.326 Pb	−94.935	1.29E+04	3.39E+04
Mo + 1.333 PbTe(g) = 0.333 Mo_3Te_4 + 1.333 Pb	−74.628	3.38E+04	5.47E+04
Mn + PbTe(g) = MnTe + Pb	−143.492	−6.22E+04	−2.60E+04
Mn + 2 PbTe(g) = $MnTe_2$ + 2Pb	−172.405	−9.76E+03	2.64E+04

References

(S1) Zhuravlev, L. T. The Surface Chemistry of Amorphous Silica. Zhuravlev Model. *Colloids Surfaces A Physicochem. Eng. Asp.* **2000**, *173* (1–3), 1–38. https://doi.org/10.1016/S0927-7757(00)00556-2.

(S2) Xia, Y.; Li, J.; Zhang, Y.; Yin, Y.; Chen, B.; Liang, Y.; Jiang, G.; Zare, R. N. Contact between Water Vapor and Silicate Surface Causes Abiotic Formation of Reactive Oxygen Species in an Anoxic Atmosphere. *Proc. Natl. Acad. Sci.* **2023**, *120* (30). https://doi.org/10.1073/pnas.2302014120.

(S3) A. Roine. HSC Chemistry [Software]. Metso: Pori 2019. https://www.metso.com/portfolio/hsc-chemistry/, last accessed on 17th August 2026.

(S4) Brooks, L. S. The Vapor Pressures of Tellurium and Selenium. *J. Am. Chem. Soc.* **1952**, *74* (1), 227–229. https://doi.org/10.1021/ja01121a059.

(S5) Azad, A. M.; Sreedharan, O. M.; Gnanamoorthy, J. B. A Novel Determination of Thermodynamic Activities of Metals in an AISI 316 Stainless Steel by a Metastable Emf Method. *J. Nucl. Mater.* **1987**, *144* (1–2), 94–104. https://doi.org/10.1016/0022-3115(87)90284-4.

(S6) Cheng, H.; Li, Z.; Leng, B.; Zhang, W.; Han, F.; Jia, Y.; Zhou, X. Intergranular Diffusion and Embrittlement of a Ni-16Mo-7Cr Alloy in Te Vapor Environment. *J. Nucl. Mater.* **2015**, *467*, 341–348. https://doi.org/10.1016/j.jnucmat.2015.09.053.

(S7) Binnewies, M.; Milke, E. *Thermochemical Data of Elements and Compounds*, 2nd ed.; Wiley, 2002.
